# Multi-Model- and Soft-Transition-Based Height Soft Sensor for an Air Cushion Furnace

**DOI:** 10.3390/s20030926

**Published:** 2020-02-10

**Authors:** Shuai Hou, Xinyuan Zhang, Wei Dai, Xiaolin Han, Fuan Hua

**Affiliations:** 1 School of Information and Electrical Engineering, Hebei University of Engineering, Handan 056038, China; houshuai@hebeu.edu.cn (S.H.); zxiny259@163.com (X.Z.); wwcxfdsz97@163.com (X.H.); 2 School of Information and Control Engineering, China University of Mining and Technology, Xuzhou 221116, China; 3 The State Key Laboratory of Rolling and Automation, Northeastern University, Shenyang 110819, China; huafa@ral.neu.edu.cn

**Keywords:** air cushion furnace, soft sensor, data-driven, transition, height prediction

## Abstract

The floating height of the strip in an air cushion furnace is a key parameter for the quality and efficiency of production. However, the high temperature and high pressure of the working environment prevents the floating height from being directly measured. Furthermore, the strip has multiple floating states in the whole operation process. It is thus difficult to employ a single model to accurately describe the floating height in different states. This paper presents a multi-model soft sensor to estimate the height based on state identification and the soft transition. First, floating states were divided using a partition method that combined adaptive k-nearest neighbors and principal component analysis theories. Based on the identified results, a hybrid model for the stable state, involving a double-random forest model for the vibration state and a soft-transition model, was created to predict the strip floating height. In the hybrid model for the stable state, a mechanistic model combined thick jet theory and the equilibrium equation of force to cope with the lower floating height. In addition, a novel soft-transition model based on data gravitation that further reflects the intrinsic process characteristic was developed for the transition state. The effectiveness of the proposed approach was validated using a self-developed air cushion furnace experimental platform. This study has important value for the process prediction and control of air cushion furnaces.

## 1. Introduction

According to statistics from the Statista research department, international car sales exceeded 80 million in 2019. While bringing convenience to production and life, cars also have a negative impact on energy, the environment, and safety. Using lightweight automobiles is a main route for guaranteeing energy-saving, environmental protection, and pedestrian safety [1]. Due to the fact that aluminum and its alloys have low density, low carbon content, and good manufacturability properties, adopting aluminum alloy instead of steel can effectively promote the development of lightweight automobiles [2].

Aluminum alloy for auto body sheets (ABSs) are the top-class aluminum alloy products owing to their better surface quality and mechanical performance. If these strips are produced using a traditional vertical furnace, some scratches and soft spots caused by fixtures may occur, resulting in poor quality [3]. An air cushion furnace is a new type of heat-treating equipment, which suspends the strip in the air for processing to prevent the strip from contacting any object such that the strip has a good surface quality. Moreover, compared with the traditional box-type heat treatment furnace, the air cushion furnace has a higher production efficiency [4]. Therefore, the air cushion furnace has been indispensable equipment in the production process of aluminum alloy sheets.

The floating height of strip is a vital factor affecting the production efficiency and quality in the air cushion furnace. First, the floating height will have a great impact on the heating rate. Second, a strip that floats too high or too low will be scratched by the equipment. Last, an aluminum alloy strip could cause a production line shutdown when it fractures. Therefore, the accurate prediction of the floating height of a strip is a key technical problem in the production process of an air cushion furnace.

In view of the importance of the strip floating height in an air cushion furnace, some scholars have studied the strip floating in an air cushion furnace. Cho pointed out that the strip has two floating states in the working process of an air cushion furnace [5]. The experimental results show that the floating characteristics of a strip is strongly affected by pressure, flow speed, and other variables. However, the stable and vibration states cannot be switched immediately, and there is a short transition between the two states. The transition state has different characteristics in different processes, showing dynamic characteristics of crossing and gradual change between the two neighboring phases. Regularly, the transition state first shows underlying features that are more similar to the previous state, and then shows those that are more similar to the next state at the end of the transition [6].

Many investigations on the floating height of a strip under a stable state have been made. Referring to the jet theory in the field of hovercraft engineering, Davie simplified the Navier–Stokes equation and derived a thin jet model [7]. More than a decade later, Chang demonstrated that a thick jet model has more advantages when a strip floats very low [8]. However, the above mechanistic model is built based on a flow profile uniform and other ideal hypotheses, neglecting some undesirable factors in the actual industrial process. It is therefore difficult to meet the requirements of a practical industrial prediction. Hou constructed a hybrid height prediction model, which combines the advantages of mechanistic and data models [9]. However, thin jet theory is still not suitable for the scene with a strip that has a low floating height [8]. The vibration floating characteristics and the instability conditions were discussed by Cho and Takeda et al. [5,10]. However, the floating process of the strip involves a fluid–solid coupling process that is too complex to be modeled. 

Soft sensor and machine learning technologies have provided more methods for problem solving. Successful applications toward the prediction of vibration signal have been made in terms of hydrology [11,12], climate [13], wind speed [14], industrial processes [15,16], and other fields. Especially, soft sensors have been widely applied to predict the industrial parameters that cannot be directly measured or can only be measured at high cost [16,17,18,19,20]. Considering the complicated process, it is difficult to build mechanistic models, thus data-driven models, such as multivariate statistical analysis [19,20], neural networks [17,21], and random forecast (RF) [18], are now introduced to develop soft sensors. 

Nevertheless, the floating height of a strip in the transition state is not taken into account in the aforementioned studies. The transition state is an inevitable dynamic process, which lasts only for a short time but has a great impact on the product quality [22]. During the transition process, the working pressure of nozzles in the air cushion furnace always varies, which will lead to a sharp fluctuation of the floating height. Furthermore, the strip is easily scratched. Hence, predicting the floating height during the transition state is of great practical significance for improving the efficiency and ensuring the quality. Hou regarded the transition state as a fuzzy region and used a hard division method to divide the floating state [23]. However, the transition state was distributed to the stable state and vibration state instead of building another model for it. Moreover, this model does not consider the scene with a very small floating height of the strip or a large nozzle opening. He et al. proposed a distributed model projection (DMP) method to cluster the nonlinear transition data and divide the transition phase into multiple sub-segments [24]. These sub-segments are considered to be steady processes and are modeled separately. Unfortunately, these sub-models are still not actually linearly dependent and they neglect dynamic characteristics of the transition state. Zhao et al. used two neighbor steady models to dynamically describe each transition state, referred to as soft-transition [25]. The weights of each model are evaluated based on distance. However, using only the distance between samples cannot fully reveal the geometric relationship between the transition and neighbor states [26,27]. The floating strip in the highest and lowest positions may contact the equipment; it is thus necessary to predict and control the maximum and minimum floating heights in the transition process in advance, thereby reducing scratching of the strip.

In consideration of the complex and multi-state characteristics of an air cushion furnace in the practical industrial processes, it is difficult to build an overall model. Generally, the industrial processes with multi-state characteristics can be divided according to different states and then formulated using corresponding monitoring models. Furthermore, principal component analysis (PCA) has been widely applied for different industrial processes [28,29,30]. However, to the best of our knowledge, the partition of the floating state and the construction of the model have not been solved regarding an air cushion furnace.

This paper proposes a multi-model soft sensor for the strip-floating height in an air cushion furnace. To improve the robustness and reduce the calculation demands, an adaptive k-nearest neighbor was first utilized to determine the basic unit in a fixed search range, which was then combined with PCA to divide the floating state of the strip. Then, a mechanistic model was proposed based on the thick jet model and equilibrium equation of force. The mechanistic model connected in parallel with the data compensation model to establish a hybrid model for stable state (HMSS). Next, a double random forest for vibration state (DRFVS) was constructed to predict the maximum value and minimum value of the strip floating height. Finally, a soft-transition model based on data gravitation was proposed by considering the distance factor, the number of local samples, and their distribution rules. 

This article is organized as follows. Section 2 introduces the structure of the air cushion furnace and basic theories of PCA and data gravitation. Section 3 details the method of state identification. The height prediction model is given in Section 4. Section 5 is devoted to the experiments and analyses of the corresponding results. Finally, in Section 6, we give our conclusions.

## 2. Preliminaries 

This section briefly describes the construction and operating principle of an air cushion furnace, followed by the general usage of PCA and the definition of data gravitation.

### 2.1. Research Background Regarding an Air Cushion Furnace

**Figure 1:**
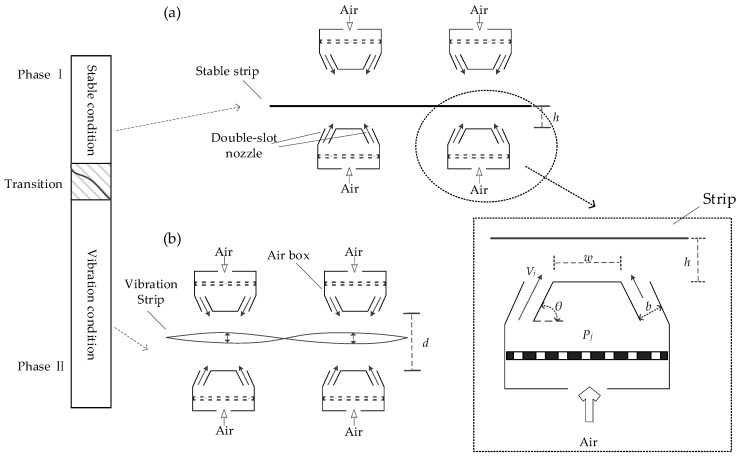
Schematic diagram of the floating process: (**a**) diagram of the stable state and (**b**) diagram of the vibration state.

An air cushion furnace is a new type of furnace. Its basic operating principle is that the strip floats in the air supported by the air flow for heating or cooling. Furthermore, the strip is required to float at a suitable height and not contact any components in the furnace. Then, the surface of strips is controlled so they are not scratched in the process, and thus, the quality can guaranteed. A simplified schematic diagram of the floating process in an air cushion furnace is shown in Figure 1.

The experimental device adopted a double slot nozzle. In Figure 1, *P_j_* is the pressure in the air box, *V_j_* is the impinging air velocity of the nozzle, *w* is the distance between the two slot nozzles, *b* is the width of a single slot nozzle, *h* is the floating height of the strip expressing the distance between the lower surface of the strip and the upper boundary of the lower nozzle, *θ* is impinging angle of the slot nozzle, and *d* is the distance between the upper nozzle and the lower nozzle. 

The working state of the floating strip mainly involves a stable state and a vibration state. The diagram of the stable state is shown in Figure 1a. In the stable state, the strip floats relatively steadily in the air and looks like a horizontal line. A diagram of the vibration state is shown in Figure 1b. In the vibration state, the strip reciprocation vibrates in a certain height range. During the vibration process, the strip is most likely to contact the equipment at the highest and lowest positions in the floating process. Moreover, the switch of the strip between stable the state and vibration states will go through a transition over a short time. The transition exhibits the gradual changes between the two different states. In this transition process, the floating state of the strip has characteristics of both the stable state and the vibration state.

### 2.2. Principal Component Analysis

As a multi-variable statistical method, principal component analysis (PCA) transforms the original data into a group of linearly independent vectors through a linear transformation, and the state information can be extracted from the complex process data [28]. The model of the PCA is shown in Equation (1):

(1) $$X=TP^{T}+E\;=\sum_{a=1}^{n}t_{a}p_{a}^{T}+E,$$

where *P* is the loading matrix, *p_a_* is load principal components (*a* = 1, 2, …, *n*), *T* is the score matrix with score vectors *t_a_* (*a* = 1, 2, …, *n*), and *n* is the number of principal components.

The squared prediction error (SPE) statistic is one method to monitor the moment of the process state change [31]. The control limit is calculated using a weighted chi-squared distribution, as seen in Equation (2):

(2) $${\mathrm{SPE}}_{\partial }\sim g\chi_{e,\partial }^{2},$$

where *g* is a weight, *e* is the number of degrees of freedom, and ∂ is the significance level.

### 2.3. Data Gravitation

In 1687, Newton published an important paper that states any two objects are attracted to each other [32]. Data gravitation models the attraction force between data using a variant of Newton’s law of gravitation [33]. Data gravitation is a scalar reflecting the similarity by comparing the gravitation between data. The similarity between two data points is also inversely proportional to the distance between them [32]. In data gravitation, the gravitation can be described using Equation (3):

(3) $$F=\frac{m_{1}m_{2}}{r^{2}},$$

where *m*_1_ and *m*_2_ are the number of samples, and *r* is distance between the centroid of two data sets. 

Peng created data particles to improving the accuracy of data gravitation calculations [34]. For any two data from the same data particles, their distances are less than this definite value and have consanguineous relation. Moreover, the gravitation effects that data particles have on other data particles can be superimposed [35].

## 3. State Identification Method

### 3.1. Adaptation K-Nearest-Neighbor-Based Data Preprocessing

For industrial processes, the noise in the collected data are inevitable, which will reduce the accuracy of the industrial state division [36]. Some scholars use the mean value of the k-nearest neighbor data as a substitute for the noise point to smooth the noise [37]. Employing nearest neighbor data as the basic unit for dividing data can effectively reduce the adverse effects of noise.

**Figure 2:**
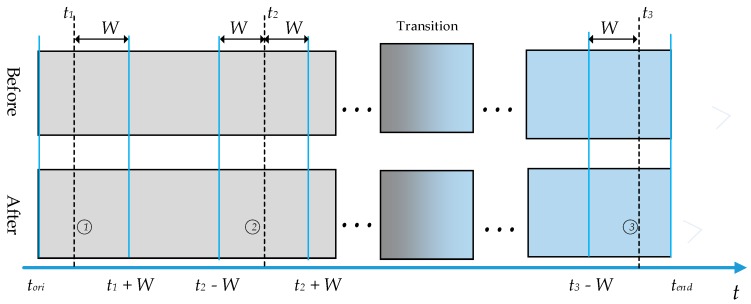
Description of the search range.

The traditional KNN method seeks samples with similar features from the whole dataset, which involves a significant computational burden. Although the nearest neighbors are similar in terms of their features, they may be discontinuous in time. Choosing such samples as nearest neighbors will affect the accuracy of the state partition. In consideration of these problems, a search range was set up in this work. As such, the KNN rule only seeks for neighbor samples in the search range, and does not consider samples outside the search range. The search range of the *i*th sample is defined in Equation (4):

(4) $$X_{i}^{t,ra}=\left\{ \begin{array}{l} X(t_{ori}:t+W)\;t<W\\ X(t-W:Q)\;t\geq W \end{array}\right.,$$

where $Q=\min\{t+W,t_{end}\}$, $t_{ori}$ is the origin sampling time of the dataset $X^{t}$, $t_{end}$ is the end sampling time of the dataset $X^{t}$. *W* is the range for searching neighbors. The settings for the search range are shown in Figure 2. In case one, the sample time $t_{1}$ of sample $x_{i}^{t_{1}}$ is between $t_{ori}$ and $t_{ori}+W$, thus the search range should be from time $t_{ori}$ to time $t_{1}+W$. In case two, the search range should be from time t−W to time t+W. In case three, the sample time $t_{3}$ of sample $x_{i}^{t_{3}}$ is between $t_{end}-W$ and $t_{end}$, thus the search range should be from time $t_{3}-W$ to time $t_{end}$.

Furthermore, the number of neighbors of a traditional KNN method is fixed to *k*. In the operation process of an air cushion furnace, if the duration of the stable state and vibration state is long, then a larger *k* value can be selected. In contrast, if the duration of the transition is short, then a smaller *k* value should be selected [24]. Sufficient neighbors may not be found in the transition state if a large *k* value is chosen. Therefore, a fixed *k* value is difficult to find that meets the requirements of three states. In order to solve this problem, an adaptive k-nearest neighbor method is adopted. Furthermore, the value of *k* is automatically determined according to the similarity between the data, rather than a fixed value. The calculation formula for the similarity is shown in Equation (5):

(5) $$sim=\frac{x_{i}^{t}\cdot x_{i,k}^{t,ra}}{\left|x_{i}^{t}\right|\cdot \left|x_{i,k}^{t,ra}\right|},$$

where the neighbors whose sim is greater than the threshold γ that constitutes neighbor data set $N_{i}=\left\{x_{i,1}^{t,ra},x_{i,2}^{t,ra}\ldots ,x_{i,k}^{t,ra}\right\}$ of $x_{i}^{t}$, k is an integer, and γ is a parameter that is set manually. The neighbor set $N_{i}$ represents $x_{i}^{t}$ as a basic unit to participate in the subsequent algorithm.

### 3.2. State Identification Algorithm

During the operation process of the air cushion furnace, the strip has three floating states: a stable state, a vibration state, and a transition between the two. Before building the model, the state needs to be divided and identified. According to the historical experience, the duration of the transition was far less than the stable and vibration states. In addition, the time series of the process data should be taken into consideration during the state partitioning. The identification method is as follows:

Input: process the dataset $\left(X,t,L\right)=\left\{\left(x_{1},t_{1},L_{1}\right),\left(x_{2},t_{2},L_{2}\right),\ldots \left(x_{i},t_{i},L_{i}\right),\ldots \left(x_{N},t_{N},L_{N}\right)\right\}$, time threshold δ, historical dataset at stable state $X_{s}$, and historical dataset at vibration state $X_{v}$. r and u are initialized to be 1.

Step 1: Sort samples (X,t,L) with the time variable in chronological order, using time as an index. Then, the dataset is normalized to $\left(X^{t},L\right)=\left\{\left(x_{1}^{t},L_{1}\right),\left(x_{2}^{t},L_{2}\right),\ldots \left(x_{i}^{t},L_{i}\right),\ldots \left(x_{N}^{t},L_{N}\right)\right\}$.

Step 2: Calculate the geometric centers of the stable state data $X_{s}$ and vibration state data $X_{v}$, termed $C_{tos}$ and $C_{tov}$, respectively.

Step 3: The neighbor set $N_{i}$ of the ith sample is calculated using the method in Section 3.1 as the basic unit of $x_{i}^{t}$.

Step 4: Extract the main feature of $x_{i}^{t}$ using the PCA method. The loading matrix can be computed using a simple eigenvalue decomposition of the sample covariance. Then, calculate the control limit of SPE for each neighbor set using a weighted chi-squared distribution, and the control limit is recorded as $Ctl_{i}$.

Step 5: The first *u* samples of $X^{t}$ make up the data segment called $Seg_{r,u}^{t}(X)$. Perform PCA on $Seg_{r,u}^{t}(X)$ to obtain the loading matrix $P_{r,i}$. Then, a residual matrix could be calculated using Equation (6):

(6) $$E_{r,i}=N_{i}P_{r,i}P_{r,i}^{T};\;$$

hence, calculate the control limit of the SPE according to $E_{r,i}$ and call it $Ctl_{r,i}$.

Step 6: Compare the current $Ctl_{r,i}$ with $Ctl_{i}$. If $Ctl_{r,i}>\alpha \cdot Ctl_{i}$ (where α is a tuning parameter), the moment of this iteration is recorded as t*; then, proceed to step 7. Otherwise, add the next neighbor set into the $Seg_{r,u}^{t}(X)$. Update u to u + 1, update i to i + 1, and return to step 5.

Step 7: The time index before t* is determined as phase r. If the duration of this phase is less than the threshold *δ*, the phase r is determined to be the transition state; then, proceed to step 9. Otherwise, go to the next step.

Step 8: Calculate the geometric center $C_{r}$ of the samples under phase r. Compute the Euclidean distances from $C_{r}$ to $C_{tos}$ and $C_{tov}$ termed $d_{r,s}$ and $d_{r,v}$, respectively. If $d_{r,s}$ > $d_{r,v}$, phase r is determine to be a stable state. Otherwise, phase r is determined to be a vibration state

Step 9: Take out the determined phase. Update r to r + 1 and set u = 1. Return to step 5 till the end of the data length.

## 4. Multi-Model Floating Height Prediction Method

### 4.1. Hybrid Model for the Stable State

#### 4.1.1. Stable State Floating Height Mechanistic Model

The strip floating height prediction model based on thin jet theory has been established. The thin jet model is often useful for the case of h/b≫1, i.e., the strip floating height far outweighs the jet thickness *b*. Nevertheless, the strip usually floats with a lower height in a stable state. In contrast, thick jet theory is more suitable for the case of h/b≤4*,* and can better predict the floating height in the stable state. Thick jet theory assumes that the profile of the aircushion layer exists as a pressure gradient field with a uniform change owing to the centrifugal force [8]. Equation (7) is the horizontal force balance for the air jet, which requires:

(7) $$V_{j}{}^{2}=\frac{p_{c}h}{\rho b(1+\cos\theta )},$$

where ρ is the air density, *b* is the width of slot nozzle, $p_{c}$ is the cushion pressure, *V_j_* is the velocity of the air jet, θ is the impinging angle, and *h* is the floating height.

Compared to other thick jet models, the pressure ratio proposed by Mair provides good predictions, both in circumstances of the floating height being lower than and higher than *b*. Even if the floating height of the strip is very low, or the thickness of the jet is wide, it can be used reasonably. The pressure ratio is shown in Equation (8):

(8) $$\frac{p_{c}}{P_{t}}=(1-e^{-2(b/h)(1+\cos\theta )}),$$

where $P_{t}$ is the effective total pressure of the air jet after the nozzle. To facilitate the construction of the equation, $p_{c}$ is simplified using a Taylor series, as shown in Equation (9):

(9) $$p_{c}=(\frac{2b(1+\cos\theta )}{h}-\frac{2b^{2}{\left(1+\cos\theta \right)}^{2}}{h^{2}}+\frac{4}{3}\frac{b^{3}{\left(1+\cos\theta \right)}^{3}}{h^{3}})P_{t}.\;$$

On the basis of Equation (9), the air cushion pressures on the lower and upper surfaces of the strip are set as $p_{c1}$ and $p_{c2}$, respectively. Then, the pressure in the lower air box is set to $P_{t1}$, and the pressure in the lower air box is set to $P_{t2}$:

(10) $$p_{c1}=(\frac{2b(1+\cos\theta )}{h}-\frac{2b^{2}{\left(1+\cos\theta \right)}^{2}}{h^{2}}+\frac{4}{3}\frac{b^{3}{\left(1+\cos\theta \right)}^{3}}{h^{3}})P_{t1},\;$$

(11) $$p_{c2}=(\frac{2b(1+\cos\theta )}{d-h}-\frac{2b^{2}{\left(1+\cos\theta \right)}^{2}}{{\left(d-h\right)}^{2}}+\frac{4}{3}\frac{b^{3}{\left(1+\cos\theta \right)}^{3}}{{\left(d-h\right)}^{3}})P_{t2},\;$$

where *d* is the distance between the upper nozzle and the lower nozzle. Equation (12) is the lift force per unit length of the air box:

(12) $$F=p_{c}w+2\rho bV_{j}{}^{2}\sin\theta ,$$

where *w* is the distance between the two slot nozzles.

Suppose that the forces applied to the lower and upper surface of the strip are *F*_1_ and *F*_2_, respectively. Then, Equation (7) is substituted into Equation (12):

(13) $$F_{1}=p_{c1}\cdot \left[w+\frac{2h\cdot \sin\theta }{1+\cos\theta }\right],$$

(14) $$F_{2}=p_{c2}\cdot \left[w+\frac{2(d-h)\cdot \sin\theta }{1+\cos\theta }\right].$$

The strip will float in the air if the lift force on the lower surface minus the lift force on the upper surface is the weight per unit length of the strip. According to the equilibrium equation of force, the balance of gravity can be written as Equation (15).

(15) $$F_{1}-F_{2}=G$$

where, *G* is the weight per unit length of the strip. Equation (16) about the strip floating height *h* is determined by combining Equations (10), (11) and (13)–(15):

(16) $$Ah^{6}+Bh^{5}+Ch^{4}+Dh^{3}+Eh^{2}+Ih^{1}+J=0$$

The predicted value *h* can be obtained by solving Equation (16). Each coefficient *A*, *B*, *C*, *D*, *E*, *F*, and *J* in Equation (16) is shown in Equations (17)–(23), respectively:

(17) $$A=4n_{2}P_{t2}b\sin\theta -4n_{1}P_{t1}b\sin\theta +G,$$

(18) $$\begin{array}{l} B=n_{1}P_{t1}[-2wb(1+\cos\theta )+12db\sin\theta +4b^{2}\sin\theta (1+\cos\theta )]-3dG\\ \begin{array}{ccc} & & +n_{2}P_{t2}[-2wb(1+\cos\theta )-12db\sin\theta +4b^{2}\sin\theta (1+\cos\theta )] \end{array} \end{array}$$

(19) $$\begin{array}{l} C=n_{1}P_{t1}[6wdb(1+\cos\theta )+2wb^{2}{\left(1+\cos\theta \right)}^{2}-12db^{2}\sin\theta (1+\cos\theta )-\frac{8}{3}b^{3}\sin\theta {\left(1+\cos\theta \right)}^{2}\\ \begin{array}{ccc} & & -12d^{2}b\sin\theta ]+n_{2}P_{t2}[-2wb^{2}{\left(1+\cos\theta \right)}^{2} \end{array}-\frac{8}{3}b^{3}\sin\theta {\left(1+\cos\theta \right)}^{2}-8db^{2}\sin\theta (1+\cos\theta )\\ \begin{array}{ccc} & & +4wdb(1+\cos\theta )+12db\sin\theta ]+3d^{2}G \end{array} \end{array}$$

(20) $$\begin{array}{l} D=n_{1}P_{t1}[-6wd^{2}b(1+\cos\theta )-6wdb^{2}{\left(1+\cos\theta \right)}^{2}-\frac{4}{3}wb^{3}{\left(1+\cos\theta \right)}^{3}+12db^{2}\sin\theta (1+\cos\theta )\\ \begin{array}{ccc} & & +8db^{3}\sin\theta {\left(1+\cos\theta \right)}^{2}+4d^{3}b\sin\theta ]+n_{2}P_{t2}[-2wd^{2}b(1+\cos\theta ) \end{array}-\frac{8}{3}db^{3}\sin\theta {\left(1+\cos\theta \right)}^{2}\\ \begin{array}{ccc} & & +2wdb^{2}{\left(1+\cos\theta \right)}^{2}-\frac{4}{3}wb^{3}{\left(1+\cos\theta \right)}^{3}-4d^{3}b\sin\theta +4d^{2}b^{2}\sin\theta (1+\cos\theta ) \end{array}]-d^{3}G \end{array}$$

(21) $$\begin{array}{l} E=n_{1}P_{t1}[2wd^{3}b(1+\cos\theta )+6wd^{2}b^{2}{\left(1+\cos\theta \right)}^{2}+4db^{3}{\left(1+\cos\theta \right)}^{3}-4d^{3}b^{2}\sin\theta (1+\cos\theta )\\ \begin{array}{ccc} & & -8d^{2}b^{3}\sin\theta {\left(1+\cos\theta \right)}^{2}] \end{array} \end{array}$$

(22) $$I=n_{1}P_{t1}[-2wd^{3}b^{2}{\left(1+\cos\theta \right)}^{2}-4wd^{2}b^{3}{\left(1+\cos\theta \right)}^{3}+\frac{8}{3}d^{3}b^{3}\sin\theta {\left(1+\cos\theta \right)}^{2}],\;$$

(23) $$J=\frac{4}{3}n_{1}P_{t1}wd^{3}b^{3}{\left(1+\cos\theta \right)}^{3}.$$

#### 4.1.2. Parallel Structure of the Hybrid Model

The mechanistic models are usually established based on a degree of ideal assumptions. However, the actual industrial operation process is not completely ideal. In addition, some processes of the air cushion furnace, such as the complex fluid–solid coupling process, are difficult to model using a mechanistic model. Therefore, there are always some errors in the prediction when using a mechanistic model. Some studies use mechanistic models to predict the main information of the floating height of the strip and use the data-driven models to compensate for the error of the mechanistic model to achieve a good prediction result [9].

**Figure 3:**
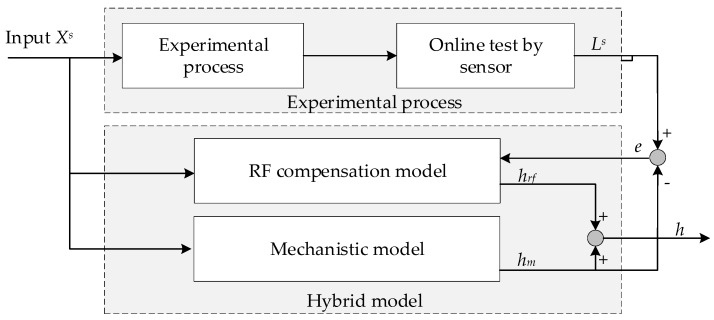
The schematic of parallel hybrid model for stable state. RF: Random forest.

The random forest (RF) algorithm has the features of clear structure, good understandability, and stronger generalization. The data quantity in our experiment was not large, which suits the RF since it has a low requirement for data quantity. Furthermore, a random forest can explain the raw model inputs and has a better prediction performance [11]. RF is employed in this work, not only because of its robust prediction capability, but also because of its transparency and flexibility of regression that could help to compensate for the error of the mechanistic model of the floating height in the air cushion furnace. In addition, the training speed of RF is relatively fast, which is beneficial for constructing a parallel structure. The parallel structure of a hybrid height prediction model is shown in Figure 3.

First, some data pairs $\left\{X^{S},L^{S}\right\}$ containing the height label were collected on the self-developed experimental platform (Air cushion furnace experimental platform, Northeastern University, Shenyang, Liaoning Province, China). In the structure shown in Figure 3, the hybrid model has two parts: the mechanistic model and the RF compensation model. On the one hand, data set $X^{S}$ is input into the mechanistic model, and this can predict main process information. The predicted value *h_m_* generated by the mechanistic model and the actual height *L* will have a certain deviation caused by partially unmodeled information, which is termed as error *e*. The error data $\left\{X^{S},e\right\}$ can be obtained. On the other hand, the RF compensation model is trained on error data $\left\{X^{S},e\right\}$. The obtained output value *h_rf_* of the RF can compensate for the difference between the actual value and the output of the mechanistic model [38]. Finally, the compensation value *h_rf_* is added to the predicted value *h_m_* to obtain the strip floating height *h* in the stable state. The hybrid model not only considers the thick jet theory and the force equilibrium equation, but also takes full advantage of the information in the data. Further, the model compensates for uncertainties that arise from the inherent process complexity.

### 4.2. Double Random Forest for Vibration State

**Figure 4:**
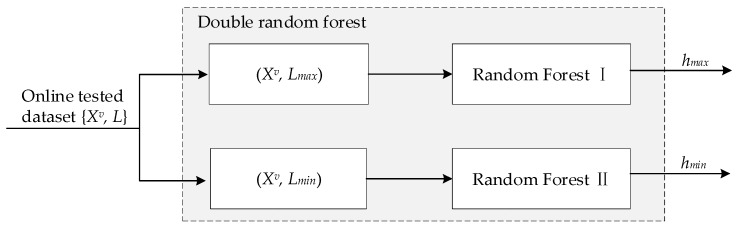
Schematic of the double random forest model for the vibration state.

In the vibration state, the surface quality will not be affected when the highest and lowest points of the floating strip have no contact with the nozzles. Therefore, the peak values could be the key variable of prediction [39,40]. An effective method to assure the surface quality is to construct a model to predict the maximum and minimum floating heights of the strip in the vibration state and controlling the process parameters to ensure the floating height is within a reasonable range. In this part, a double random forest model is proposed to predict the maximum and minimum floating height in the vibration state. The schematic of the prediction model is shown in Figure 4.

In accordance with Figure 4, several experiments were carried out based on this self-developed experimental device. Groups of height sampling data were collected online by multiple sensors in the vibration state to form the experimental data set *X^v^*. The strip floating height in the vibration state was measured and recorded with a laser rangefinder. MATLAB (2017a) (MathWorks, Natick, MA, USA) was used to extract the maximum values *L_max_* and minimum values *L_min_* in each group of data. The data set {*X^v^*, *L_max_*} with the maximum value label and the data set {*X^v^, L_min_*} with the minimum value label were used to train a random forest model each. The double random forest was implemented using MATLAB (2017a, Toolkit: RF_MexStandalone-v0.02).

### 4.3. Soft-Transition Prediction Model

As is mentioned above, a parallel hybrid model for the stable state and double random forest model for the vibration state were established. Additionally, there is an intermediate state between the two states, that is, the transition. In the transition stage, the floating process of the strip shows dynamic characteristics of crossing and gradual change with the two neighboring states. The two nearest-neighbor states refer to the stable and vibration states connecting with the transition head and tail. In fact, a single stable prediction model or vibration prediction model cannot accurately describe such a crossing and gradually changing dynamic characteristics [41]. In order to predict the height of strip floating in the transition state, a novel soft-transition modeling method is proposed in this paper.

The soft-transition strategy uses two models of neighbor states to dynamically describe each transition. The proportion of two models is automatically determined by the characteristic correlation between the current transition and its two neighbor states, which is called the membership degree. In this work, data gravitation is used as the basis for calculating the membership degree, which further reflects the inherent geometrical properties of the dataset. Data gravitation takes not only the distance relationship between the data, but also the distribution and density into account, which can better express the correlation between a single datum and a cluster of data [34]. 

Suppose that a data set of a neighbor state has *n* particles, termed $X_{1}^{p},X_{2}^{p},\ldots X_{i}^{p},\ldots X_{n}^{p}$. The number of samples in the data particle $X_{i}^{p}=\{x_{i1}^{p},x_{i2}^{p},\ldots x_{ij}^{p},\ldots x_{im_{i}}^{p},\}$ is $m_{i}$. Then, the geometric center of $X_{i}^{p}$ can be calculated using Equation (24):

(24) $$x_{i}^{c}=\frac{\sum_{j=1}^{m_{i}}x_{ij}^{p}}{m_{i}},j=1,2,\ldots ,m_{i}.\;$$

Then, for a sample, the composition of gravitation of one state is given using Equation (25):

(25) $$F_{g}=\sum_{i=1}^{n}\frac{m_{i}}{r_{i}^{2}},\;$$

where $r_{i}$ is the Euclidean distance from this sample to $x_{i}^{c}$.

For a sample $x_{i}^{c}$ in the transition stage, two data particle sets are established according to the stable state and vibration state lying at both ends of the transition. Then, the composition of the data gravitation from the stable data particle set and the vibration data particle set to $x_{i}^{t}$ is calculated, respectively, and are termed *F_s_* and *F_v_*. For a transition, the membership degree of the stable state is $w_{s}$, and the membership degree of vibration state is $w_{v}$. The expressions are as given as Equations (26) and (27):

(26) $$w_{s}=\frac{F_{s}}{F_{s}+F_{v}},\;$$

(27) $$w_{v}=\frac{F_{v}}{F_{s}+F_{v}}.\;$$

Inputting $x_{i}^{t}$ into the hybrid model in Section 4.1 gives the output *h_s_*. Inputting $x_{i}^{t}$ into the double random forest in Section 4.2 gives the outputs *h_v_*_max_ and *h_v_*_min_. The maximum value and minimum value of the strip floating height in the transition prediction model are shown in Equations (28) and (29):

(28) $$h_{\mathrm{tmax}}=w_{v}h_{v\max}+w_{s}h_{s},$$

(29) $$h_{\mathrm{tmin}}=w_{v}h_{v\min}+w_{s}h_{s}\;.$$

### 4.4. Multi-Model Floating Height Soft Sensor

**Figure 5:**
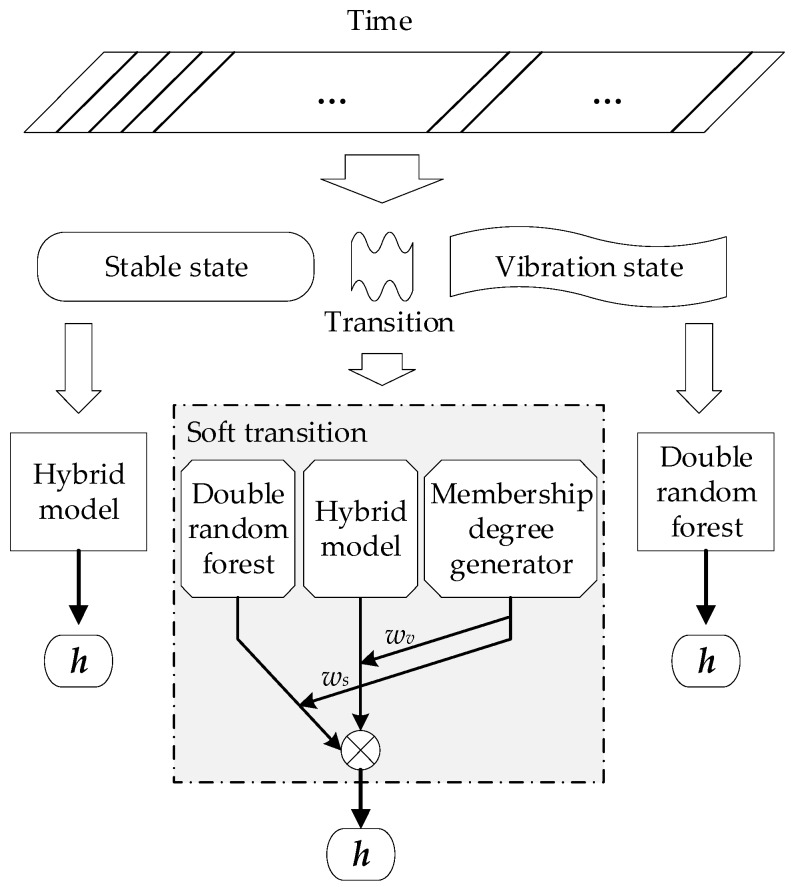
Schematic of the multi-model floating height soft sensor.

Given that there are three possible states for the strip floating in an air cushion furnace, the process characteristics of a transition may be quite like the two neighbor states but dynamic. In this paper, a multi-model floating height soft sensor based on state identification is proposed. As shown in Figure 5, the state of the process data is first divided into the stable state, vibration state, and transition state. For samples in the stable state, the hybrid model of mechanistic model and random forest given in Section 4.1 is used for prediction. For samples in the vibration state, the double random forest model given in Section 4.2 is employed to predict the maximum value and minimum value of the floating height. For samples in the transition state, a stable prediction mode is used in conjunction with the vibration prediction model according to the membership degrees $w_{s}$ and $w_{v}$. The membership degrees are determined using a soft-transition method.

## 5. Illustration and Discussion

**Figure 6:**
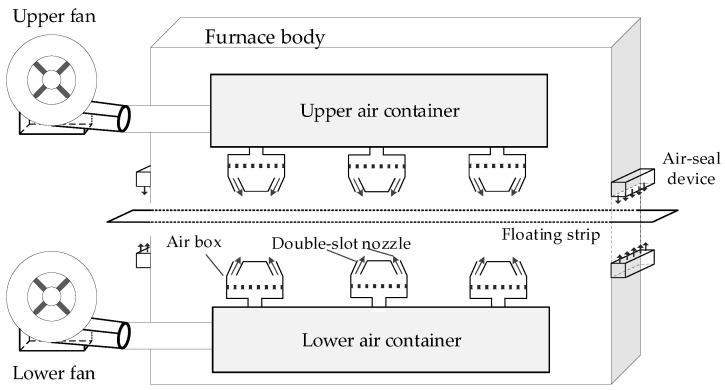
The schematic diagram of an air cushion furnace.

**Figure 7:**
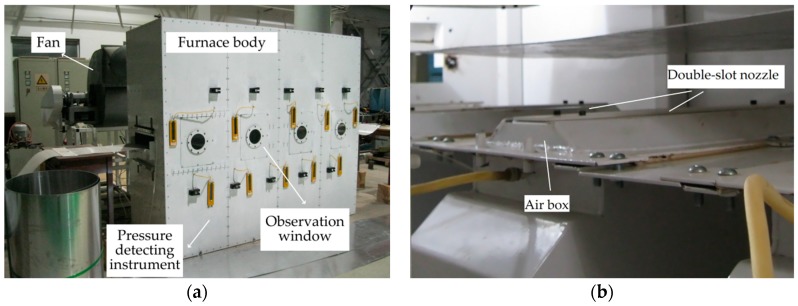
Equipment of an air cushion furnace experimental platform: (**a**) external view of the equipment and (**b**) interior view of the equipment.

The proposed approach was tested in the self-developed air cushion furnace experimental platform. The specification of the experimental platform is as stated in Hou et al. [23]. In the production process, the floating height could not be continuously measured by sensors due to the technical limitation. On the experimental platform, we made an observation window to temporarily detect data through the sensors [9]. The schematic diagram of the air cushion furnace experimental platform is shown in Figure 6. Figure 7 shows the equipment of an air cushion furnace. 

The width of the strip in our experiment was 300 mm. A total of 2200 test samples were collected during the operation process of the air cushion furnace. Among the tests, there were 630 stable state samples, 1420 vibration state samples, and 150 transition state samples. It is well known that white noise is inevitable in practical industrial processes, and the white noise can be regarded as an infinite order persistent excitation signal. The persistent excitation thus always exists.

The root mean square error (RMSE) and mean absolute error (MAE) are scoring rules that represent the average magnitude of the error. Lower values of RMSE and MAE stand for more efficient models (zero for the perfect model). Here, the RMSE and MAE were taken as evaluation standards of performance of the models, where the equations are shown as Equations (31) and (32):

(30) $$\mathrm{RMSE}=\sqrt{\frac{1}{N}\sum_{i=1}^{N}{\left(h_{i}-y_{i}\right)}^{2}},\;$$

(31) $$\mathrm{MAE}=\frac{1}{N}\sum_{i=1}^{N}|h_{i}-y_{i}|,\;$$

where *N* is the number of samples, $h_{i}$ is the actual height, and $y_{i}$ is the prediction value of strip floating height.

The validity of the proposed method was proved using three steps. First, the proposed state identification method was verified on four sets of data regarding the strip floating state. The state of the process data was divided into the stable state, vibration state, and transition state. Furthermore, the divided data was used to demonstrate the effectiveness of the proposed hybrid model in the stable state and double random forest in the vibration state. Finally, strips with two thicknesses were used to describe the effect of the soft transition.

### 5.1. Validation of the State Identification Method

**Figure 8:**
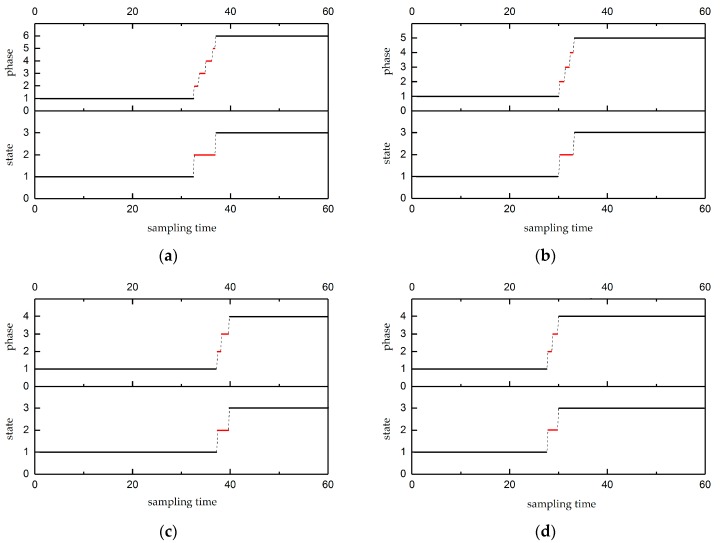
State divided and transition state identification results: (**a**) thickness = 1.5 mm, upper pressure = 40 Pa; (**b**) thickness = 1.5 mm, upper pressure = 90 Pa; (**c**) thickness = 2 mm, upper pressure = 40 Pa; and (**d**) thickness = 2 mm, upper pressure = 90 Pa.

The strip floating process was divided into three states, including the stable state, the vibration state, and the transition state. In this work, the nozzle pressures of the two strip thicknesses were set at different values, producing four groups for the strip floating state. The identification results are shown in Figure 8.

In Figure 8, because of the dynamic characteristics of the transition state, the transition state (indicated with red lines) was divided into several phases during the running process of the algorithm, as shown in top of Figure 8a–d. These phases were finally identified as three floating states using a subsequent algorithm, as shown in the bottom of Figure 8a–d, where states 1–3 represent the stable state, transition state, and vibration state, respectively.

For example, in the top of Figure 8a, the whole working process was divided into six phases. The duration time of the first phase was 32.4 s; the duration times of the second to fifth phases were 0.8 s, 1.2 s, 1.2 s, and 0.4 s, respectively; and the duration time of the last phase was 23 s. It is obvious that the duration of phases 2–5 were far less than phases 1 and 6. Then, the transition was identified and merged using threshold *δ* for the duration time. The results are given in the bottom of Figure 8a. The stable state was found from the sampling time of 0–32.4 s, the transition state was from the sampling time of 32.6–36.8 s, and the vibration state was from the sampling time 37–60 s. It can also be found in Figure 8b–d that the duration of the intermediate states was much less than the first and last states, and they were identified as transition states using the threshold *δ* of the duration time. In Figure 8, state 1 was a stable state, state 2 was a transition state, and state 3 was a vibration state. The results show that the stable state, vibration state, and especially the transition state were successfully identified. Hence, the proposed method was effective for dividing and identifying the floating state of the strip. 

Based on the identification results, the multi-model floating height prediction method was carried out by using divided data. The corresponding illustrations of the hybrid prediction model for the stable state, double random forest model for the vibration state, and soft-transition prediction model are discussed in Section 5.2 and 5.3. 

### 5.2. Validation in the Stable and Vibration States

**Figure 9:**
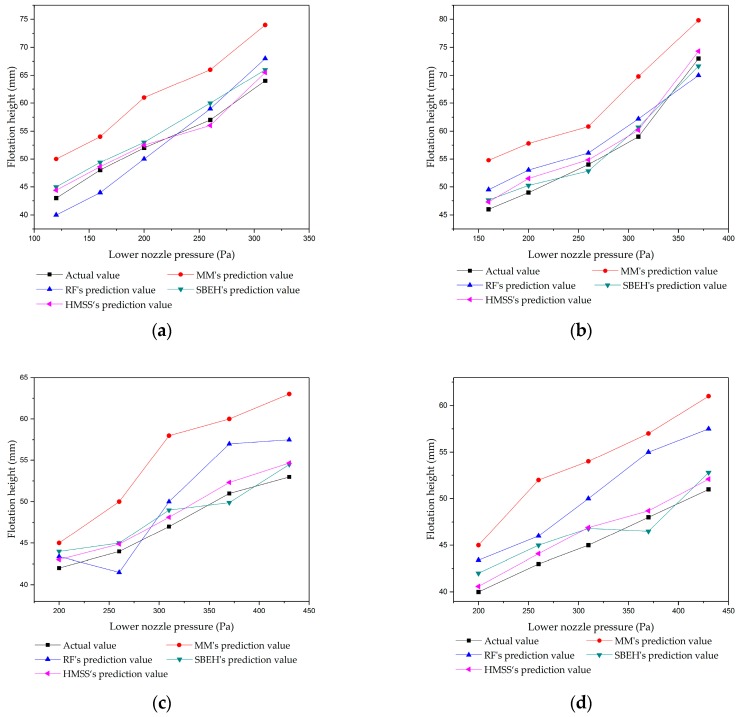
The prediction and actual floating height in the stable state: (**a**) thickness = 1.5 mm, upper pressure = 40 Pa; (**b**) thickness = 1.5 mm, upper pressure = 90 Pa; (**c**) thickness = 2 mm, upper pressure = 40 Pa; and (**d**) thickness = 2 mm, upper pressure = 90 Pa. RF: Random forest, HMSS: Hybrid model for stable state, MM: mechanistic model, SBEH: Hybrid selective bagging ensemble model.

**Figure 10:**
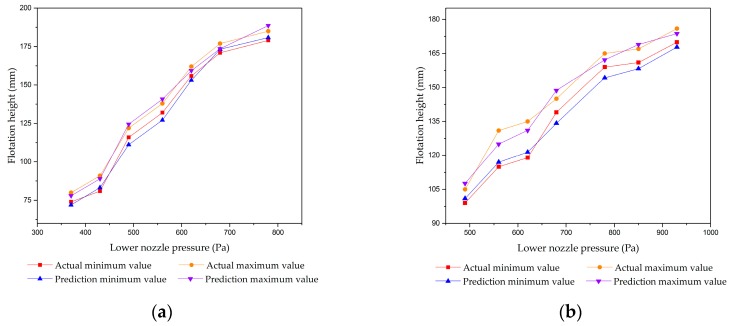

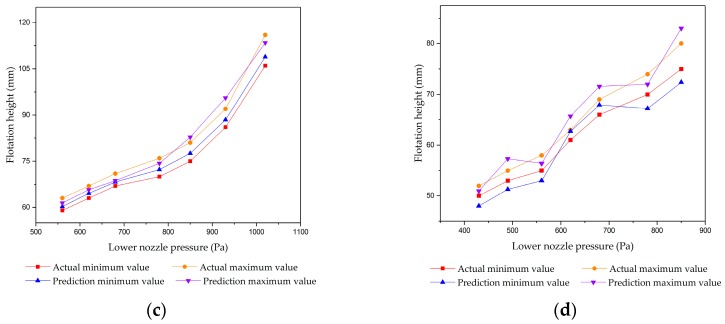
The maximum and minimum floating height at vibration state: (**a**) thickness = 1.5 mm, upper pressure = 40 Pa; (**b**) thickness = 1.5 mm, upper pressure = 90 Pa; (**c**) thickness = 2 mm, upper pressure = 40 Pa; and (**d**) thickness = 2 mm, upper pressure = 90 Pa.

The floating heights of the two thicknesses of strips were measured at different lower and upper nozzle pressures. For the stable prediction model, the RF method, the mechanistic model, and the hybrid selective bagging ensemble model (SBEH) [9] were performed together with the proposed method. Figure 9 shows the experimental results in the stable state. The conditions of the thicknesses of the strips were 1.5 mm and 2 mm, and the upper nozzle pressures were fixed at 40 Pa and 90 Pa, respectively. The predicted minimum value and maximum value in the vibration state are shown in Figure 10. 

The evaluation results, including MAE and RMSE, obtained using the HMSS and the other three benchmark models are given in Table 1 and Table 2. We provide the MAE and RMSE of the DRFVS in Table 3.

Table 1 and Table 2 and Figure 9 show that the HMSS had the lowest total RMSE and total MAE. This means that the predicted results of the algorithm in this paper were more consistent with the actual results than other algorithms. Furthermore, the algorithm showed a good prediction result. Among the above models, the mechanistic model had the worst prediction result. This was because the mechanistic model is based on a certain ideal hypothesis and neglects some undesirable factors in the actual industrial process. The unmodeled part of the mechanistic model will produce some structural errors. The prediction performance of RF was better than the mechanistic model (MM). The RF model could express a highly nonlinear relation, which had a better effect than polynomial fitting. Thus, a RF is an effective tool for use as a soft sensor. Compared with MM and RF, the prediction effect of the two hybrid models was obviously better. This was due to the RF compensating for the unmodeled part of the mechanistic model, and the mechanistic model enhancing the generalization and interpretability of the RF. On the other side, HMSS was slightly better than SBEH. The main reason was that the floating height of the strip was usually lower in the stable state. The mechanistic model in SBEH is based on thin jet theory, while the mechanistic model in HMSS is based on thick jet theory, which is more suitable for the situation of a low floating height in a stable state. 

According to the experimental results in the vibration state, using a double random forest to predict the maximum value and the minimum value of the floating height obtained a satisfactory result.

### 5.3. Demonstration of the Transition State

**Figure 11:**
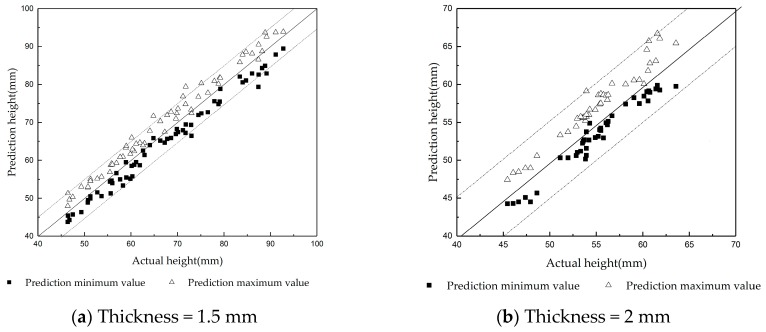
The performance of the floating height prediction in the transition state. The abscissa of the solid block represents the actual value of the strip floating height and the ordinate is the corresponding predicted minimum value of the soft-transition model. The hollow triangle appears in pairs with the solid block. The abscissa represents the actual value of the same strip floating height and the ordinate is the corresponding predicted maximum value of the soft-transition model.

The proposed soft-transition method was tested on strips with 1.5 mm and 2 mm thicknesses. During the floating process of the 1.5 mm strip, when the upper nozzle pressure was fixed to 0, 5, 10, 60, 80, 90, 120, 160, 200, 260, 310, and 430 Pa in turn, the lower nozzle pressure increased gradually from 40 Pa. Furthermore, the transition data was collected under 12 experimental conditions. During the floating process of the 2 mm strip, when the upper nozzle pressure was fixed to 5, 10, 40, 60, 80, 90, and 120 Pa in turn, the lower nozzle pressure increased gradually from 40 Pa. Furthermore, the transition data was collected under seven experimental conditions. The contrast among the prediction maximum value, the prediction minimum value, and the actual height are shown in Figure 11.

In order to visually express the relationship between the predicted result and the actual height, three auxiliary lines are placed in Figure 11. The point above the solid line indicates that the predicted value was greater than the actual height, and the point below the solid line indicates that the predicted value was less than the actual height. Two auxiliary dotted lines are drawn at a pitch of deviation 5 mm above and below the solid line, respectively. The region between the two dotted lines indicates that the error between the predicted and the actual height was in the range of ±5 mm. The region outside the two dotted lines indicates that the error between the predicted value and the actual height was greater than 5 mm.

For expedient understanding, the statistical results are shown in Table 4, which list the hit rate of the actual height greater than prediction minimum value, as well as less than the prediction maximum value. That is, the ratio of the number of sample points whose actual height was between predicted maximum value and the predicted minimum value to the total number of sample points is called the qualitative hit rate. The ratio of the number of sample points whose predicted maximum value and predicted minimum value were within a distance of ±5 mm at the true height to the total number of sample points is called the quantitative hit rate. The qualitative hit rate reflects the effectiveness and accuracy of the prediction of the maximum value and minimum value. The quantitative hit rate can provide a more accurate range and referential value for the prediction of the strip floating height.

From Figure 11 and Table 4, it is seen that the probability of the actual height being less than the prediction maximum value and greater than prediction minimum value, i.e., the qualitative hit rate, was higher than 90 percent. Moreover, values predicted by the soft-transition model were mainly in the range of ±5 mm of the actual height. Therefore, using the data-gravitation-based soft-transition model to predict the maximum and minimum values of the strip floating height can play a role in the guarantee of product quality in the production process.

## 6. Conclusions

The high-quality metal strip produced by air cushion furnace is an important part for automobiles and satellites, which affects the economy and peoples’ livelihoods. In order to predict the key parameter that is the floating height of strip, a multi-model soft sensor under multiple states was proposed. First, the state identification algorithm, which can treat the transition state well, provided a new way for multiple state processes. Second, because the floating height of the strip is often low in the stable state, a mechanistic model was presented by employing thick jet theory and the equilibrium equation of force. Furthermore, the parallel hybrid structure combined with a data compensation model improved the prediction accuracy in the stable state. Next, the range of the floating height of the vibration state was given by constructing the double random forest model. Finally, the soft-transition model based on data gravitation further reflected the intrinsic dynamic process characteristics during the transition. Thus, the overall soft sensor could predict the floating height of the strip in any of the three floating states. The feasibility and effectiveness of the proposed soft sensor was demonstrated on the self-developed air cushion furnace experimental platform. The content of this paper has important research value and practical significance for the high-efficiency and high-quality production of the air cushion furnace. Also, parameter forecasting widely exists in industrial processes and the approach provides new ideas and reference value for other multiple-state industrial processes.

## Figures and Tables

**Table 1:** Root mean square error (RMSE) values of different algorithms in the stable state.

Pressure (Pa)	Strip Thickness = 1.5 mm		Strip Thickness = 2 mm		Total
	40 Pa	90 Pa	40 Pa	90 Pa	
Random forest	3.131	3.222	3.833	5.231	15.417
SBEH	1.998	1.443	1.579	1.829	6.849
MM	8.331	8.532	8.331	8.529	33.723
HMSS	1.079	1.543	1.235	1.173	5.030

**Table 2:** Mean absolute error (MAE) value of different algorithms in the stable state.

Pressure (Pa)	Strip Thickness = 1.5 mm		Strip thickness = 2 mm		Total
	40 Pa	90 Pa	40 Pa	90 Pa	
Random forest	3	3.16	3.480	4.98	14.62
SBEH	1.88	1.425	1.520	1.82	6.645
MM	8.2	8.4	7.800	8.4	32.8
HMSS	1.000	1.435	1.204	1.08	4.719

**Table 3:** Prediction results of the maximum and minimum height in the vibration state.

Pressure (Pa)	Strip Thickness = 1.5 mm				Strip Thickness = 2 mm			
	RMSE		MAE		RMSE		MAE	
	40 Pa	90 Pa	40 Pa	90 Pa	40 Pa	90 Pa	40 Pa	90 Pa
Maximum Value	2.793	3.563	2.729	3.329	2.241	2.269	2.114	2.176
Minimum Value	3.193	3.185	2.971	2.971	2.143	2.138	2.057	2.100

**Table 4:** Statistics of the model performances in the transition state.

Strip Thickness	Number of Fixed Groups of Lower Nozzle Pressure	QualitativeHit Rate	QuantitativeHit Rate
1.5 mm	12	93.3%	88.3%
2 mm	7	95.2%	95.8%
